# Dr. Lewis A. Denton

**Published:** 1910-06

**Authors:** 


Dr. Lewis A. Denton, of Buffalo, died at his home in this city, May 8, 1910, aged 60 years. He graduated in medicine from Long Island College Hospital in 1876. He is survived by his wife, Dr. Mary A. Denton.

